# A phase I clinical study of a cocktail vaccine of Wilms’ tumor 1 (WT1) HLA class I and II peptides for recurrent malignant glioma

**DOI:** 10.1007/s00262-018-2274-1

**Published:** 2018-11-14

**Authors:** Akihiro Tsuboi, Naoya Hashimoto, Fumihiro Fujiki, Soyoko Morimoto, Naoki Kagawa, Hiroko Nakajima, Naoki Hosen, Sumiyuki Nishida, Jun Nakata, Satoshi Morita, Junichi Sakamoto, Yusuke Oji, Yoshihiro Oka, Haruo Sugiyama

**Affiliations:** 1 Department of Cancer Immunotherapy, Osaka University Graduate School of Medicine, 2-2 Yamada-Oka, Suita, Osaka 565-0871 Japan; 2 Department of Neurosurgery, Kyoto Prefectural University of Medicine Graduate School of Medical Science, Kyoto, Japan; 3 Department of Cancer Immunology, Osaka University Graduate School of Medicine, Osaka, Japan; 4 Department of Neurosurgery, Osaka University Graduate School of Medicine, Osaka, Japan; 5 Department of Cancer Stem Cell Biology, Osaka University Graduate School of Medicine, Osaka, Japan; 6 Department of Respiratory Medicine and Clinical Immunology, Osaka University Graduate School of Medicine, Osaka, Japan; 7 Department of Biomedical Statistics and Bioinformatics, Kyoto University Graduate School of Medicine, Kyoto, Japan; 8 Tokai Central Hospital, Kakamigahara, Japan; 9 Department of Functional Diagnostic Sciences, Osaka University Graduate School of Medicine, Osaka, Japan; 10 Department of Immunopathology, WPI Immunology Frontier Research Center, Osaka University, Osaka, Japan

**Keywords:** WT1, Peptide vaccine, Cancer vaccine, Cancer immunotherapy, HLA class II peptide, Malignant glioma

## Abstract

**Purpose:**

The safety and clinical efficacy of WT1 human leukocyte antigen (HLA) class I peptide vaccine have been established, but the safety of a cocktail vaccine of WT1 HLA class I and II peptides has not. To verify its safety, we performed a phase I clinical trial for patients with recurrent malignant gliomas and assessed the immunological responses and survival data.

**Patients and methods:**

Fourteen HLA-A*24:02-positive patients with recurrent malignant glioma (2 with grade 3, 12 with grade 4) were enrolled. Every week, the patients received alternately a vaccine containing 3 mg of WT1 HLA-A*24:02-restricted (HLA class I) peptide and a cocktail vaccine of the HLA class I peptide and one of 0.75, 1.5 or 3 mg of the WT1 HLA class II peptide. For patients who showed no significant adverse effects within 6 weeks, the WT1 vaccine was continued at 2–4-week intervals.

**Results:**

Eleven of the 14 patients completed WT1 vaccination for 6 weeks, while 3 patients dropped out earlier due to disease progression. All patients showed grade I level of skin disorders at the injection sites. No grade III/IV toxicity or dose-limiting toxicity was observed for any dose of WT1 HLA class II peptide. Six of the 14 patients had stable disease at 6 weeks. Median OS and 1-year OS rates were 24.7 weeks and 36%, respectively.

**Conclusion:**

The safety of a cocktail vaccine of WT1 HLA class I and II peptides for malignant gliomas was verified. This vaccine is, therefore, considered promising for patients with recurrent malignant glioma.

## Introduction

Prognosis of recurrent glioblastoma multiforme (GBM) is extremely poor and treatment options are limited. Extensive research to develop new treatments for recurrent GBM is ongoing [1–3], and immunotherapies have proven to be promising.

Immunotherapies for malignant gliomas have been tried in the form of several procedures such as monoclonal antibody therapy, tumor-associated antigen (TAA) peptide-based dendritic cell or vaccine immunotherapies, and checkpoint inhibitors [4–10]. Advancements in tumor immunology have resulted in the identification of TAAs that can be used for cancer vaccines, in which the TAA-derived epitopes are combined with HLA class I molecules, followed by recognition by CD8^+^ cytotoxic T lymphocytes (CTLs). One of the identified TAAs was the product of Wilms’ tumor gene 1(WT1) [11–14].

The wild-type WT1 gene has been shown to be overexpressed in hematological malignancies and almost all solid tumors [15, 16], while gliomas, as well as other solid tumors, have been found to express the WT1 protein [17]. A definite correlation has been established between WT1 expression and cellular proliferation activity as assessed by WHO grade [18]. The wild-type WT1 gene functions as an oncogene [15, 16], which suggests that tumor escape via downregulation of WT1 is unlikely to occur.

We previously performed a phase I/II clinical trial to examine the safety of as well as the clinical and immunological responses to a WT1 HLA class I peptide vaccine for HLA-A*24:02-positive patients with myelodysplastic syndrome (MDS), acute myeloid leukemia (AML), and breast and lung cancers [19, 20]. We were able to demonstrate that the WT1 peptide vaccine emulsified with Montanide ISA51 adjuvant was safe for patients with normal hematopoiesis, and that the induction of WT1 peptide-specific CD8^+^ CTLs was associated with clinical response. Furthermore, we performed a phase II clinical trial of the same vaccine for HLA-A*24:02-positive patients with recurrent glioblastoma multiforme (GBM) and again showed that it was safe with more favorable clinical outcomes than obtained with previously reported approaches for recurrent GBM [21].

CD4^+^ T cells, which recognize antigenic peptides in the context of major histocompatibility complex (MHC) class II molecules, have a helper function for priming, regulating, and maintaining CD8^+^ CTLs [22], and for guiding them to tumor sites, where they can directly kill tumor cells in an MHC class II-restricted manner [23]. One of the reasons for the transient clinical effect of HLA class I peptide vaccine may be that the CD8^+^ CTLs elicited by the HLA class I peptide could not be sufficiently stimulated by the peptide-specific CD4^+^ helper T cells and thus failed to become memory-type CTLs [22]. Simultaneous induction of the TAA-specific CTLs and CD4^+^ helper T cells is, therefore, thought to be crucial for the achievement of sufficient clinical effect.

We previously identified a WT1 protein-derived 16-mer HLA class II peptide, WT1_332_ (KRYFKLSHLQMHSRKH), which can elicit WT1_332_-specific CD4^+^ T-cell responses, and demonstrated that this WT1_332_ peptide was endogenously generated from the WT1 protein in WT1-expressing tumor cells. Dendritic cells were found to take up apoptotic WT1-expressing tumor cells, and present the WT1_332_ peptide on their cell surface in association with HLA class II molecules [24–26]. Furthermore, the WT1_332_ peptide could induce WT1_332_-specific T-helper cell type 1(Th1)-type CD4^+^ T cells, resulting in an increase in WT1-specific CD8^+^ CTLs in vitro. Importantly, WT1_332_-specific CD4^+^ T cells could not only aid the expansion of WT1-specific CD8^+^ CTLs but also directly kill HLA class II-positive WT1-expressing tumor cells [26]. In a recent phase I clinical study of the WT1_332_ peptide vaccine (OCV-501), OCV-501 containing 0.3, 1.0, or 3.0 mg of the peptide was subcutaneously administered on a weekly basis to elderly patients with AML in complete remission for 4 weeks. This study demonstrated that OCV-501 was safe and well tolerated, and that WT1_332_ peptide-specific delayed-type hypersensitivity occurred as an immunological response [27].

We designed a phase I clinical study to investigate the dose-limiting toxicity of the WT1 class II peptide in a cocktail vaccine of WT1 HLA class I and class II peptides. In this article, we report on the safety of this cocktail vaccine for patients with recurrent malignant glioma, as well as on WT1-specific immunological responses and clinical outcomes.

## Clinical materials and methods

### Patient population

Patients from 20 to 80 years old with malignant glioma were eligible for enrolment if they were resistant to conventional chemotherapy. Other inclusion criteria were: (1) immunohistochemically determined positive WT1 expression in glioma cells; (2) the presence of HLA-A*24:02 and at least one of HLA-DRB1*04:05, *15:01, *15:02, *08:03, HLA-DPB1*09:01, and *05:01; (3) performance status of 0–2 (Eastern Cooperative Oncology Group); (4) no severely impaired organ function; (5) no administration of chemotherapy, immunotherapy, immunosuppressive therapy, or radiotherapy within 4 weeks prior to WT1 vaccination.

### WT1 peptides

**Fig. 1:**
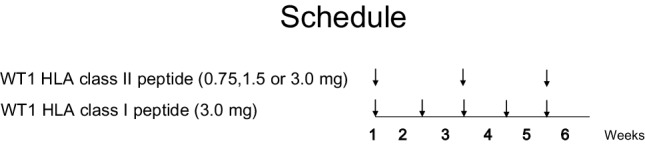
Schedule of WT1 peptide vaccination

A cocktail vaccine consisting of a mixture of HLA-A*24:02-restricted modified WT1-peptide (WT1_235_: amino acids 235–243; CYTWNQMNL, abbreviated as WT1 HLA class I peptide) [28] and HLA class II-restricted WT1 peptide (WT1_332_: amino acids 332–347; KRYFKLSHLQMHSRKH, abbreviated as WT1 HLA class II peptide) emulsified with Montanide ISA 51 adjuvant was used as the WT1 peptide vaccine (Fig. 1).

HLA-A*2402-restricted, modified 9-mer WT1-peptide was substituted for methionine (M) to tyrosine (Y) at amino acid position 2 (the anchor position) of the natural WT1 peptide (CMTWNQMNL). This modification resulted in a much stronger CTL activity against WT1-expressing tumor cells than that of the natural peptide [28]. The WT1 peptides (GMP grade) were purchased from Neo MPS Inc. (San Diego, CA, USA) or the Peptide Institute (Osaka, Japan) in the form of lyophilized peptides.

### Vaccination design

The WT1 HLA class II peptide was dose escalated from 0.75 to 1.5 and then to 3.0 mg/body while the doses of the WT1 HLA class I peptide remained at 3.0 mg. The three plus-three cohort design was used. Patients received an intradermal administration of a cocktail vaccine comprising 3 mg of WT1 HLA class I peptide and one of 0.75, 1.5 or 3.0 mg of WT1 HLA class II peptide as the vaccine, followed by a vaccine containing 3 mg of WT1 HLA class I peptide in the second week. Administration of the cocktail vaccine and the WT1 HLA class I peptide vaccine was alternated every week (Fig. 1).

The observation period for determination of safety was set at 6 weeks from the first administration of the WT1 vaccine. If the safety was confirmed during this period, WT1 vaccination was continued at 2–4-week intervals with the patients’ informed consent.

### Endpoints

The primary endpoint was safety. Toxicities were evaluated according to the Common Terminology Criteria for Adverse Events (CTCAE) version 3.0. Dose-limiting toxicity (DLT) was defined as any of the following adverse events occurring during the 6-week period from the start of the vaccination: grade 4 hematological toxicity lasting more than 7 days, grade 3 or worse neutropenia accompanied by high fever (> 38 °C) (febrile neutropenia), and any non-hematological toxicity of grade 3 or worse in other organ systems, including reactions at the vaccine injection sites. These adverse events were then judged as possibly, probably, or definitely related to treatment.

Secondary endpoints were overall survival (OS) time and WT1-specific immunological responses.

### WT1-specific immunological response

#### DTH

For immunological monitoring, delayed-type hypersensitivity (DTH) to either WT1 HLA class I or II peptides was examined by intradermally injecting 0.05 ml of a saline solution of either WT1 HLA class I or II peptide (200 µg/ml). A positive test result was defined as > 2 mm induration and/or eruption within 48 h.

#### Tetramer assay

Frozen peripheral blood mononuclear cells (PBMCs) from patients were thawed, incubated for 1 h at 37 °C in X-VIVO 15 medium (Lonza Inc., Walkersville, MD) supplemented with 10% AB serum (Gemini Bio-Products, West Sacramento, CA), and then passed through 40-µm nylon mesh to remove debris.

The cells were then incubated with Clear Back (MBL Co. Ltd., Nagoya, Japan) in phosphate buffered saline containing 5% FBS and 0.02% sodium azide (fluorescence-activated cell sorting (FACS) buffer) at room temperature for 5 min, stained with PE-labeled HLA-A*24:02/WT1_235_ mutant tetramer (WT1_235_ tetramer) (MBL) for 1 h at 4 °C, and then further stained with anti-CD3, -CD8 and -CD4 antibodies for 25 min at 4 °C in the dark. Next, the cells were washed three times, re-suspended in an appropriate volume of FACS buffer, incubated with 7-AAD (eBioscience, San Diego, CA) for 5 min before analysis, and analyzed with FACSAria (BD Biosciences, Franklin Lakes, NJ). The data were analyzed with FlowJo software (Tree Star, Ashland, OR). The frequency of WT1-specific T cells was calculated as WT1-tetramer^+^ CD8^+^ T cells/CD8^+^ T cells.

The monoclonal antibodies (mAbs) used for flow cytometric analysis were anti-CD3-Pacific Blue, anti-CD3-V500, anti-CD4-V500, anti-CD4-APC-H7, anti-CD8-FITC (Beckman Coulter, Brea, CA), anti-TNFα-APC and anti-IFNγ-PE (eBioscience).

#### Tetramer and intracellular cytokine staining assays

PBMCs were suspended in 2 ml of X-VIVO 15 medium supplemented with 10% AB serum, 40 IU/ml of human recombinant IL-2 (kindly provided by Shionogi & Co., Ltd., Osaka, Japan), 1 µg/ml of wild-type WT1_235_ peptide and 20 µg/ml of WT1_332_ helper peptide, plated into a 24-well plate, cultured for 1 week and then used for tetramer and intracellular cytokine staining assays. Statistical analysis was performed by means of the Mann–Whitney’s *U* test.

#### WT1 peptides-specific intracellular cytokine staining assay

PBMCs were incubated with 1 µg/ml of WT1 HLA class I peptide or 10 µg/ml of WT1 HLA class II peptide in the presence of 2 µg/ml CD28/CD49d Costimulatory Reagent (BD Biosciences) and 10 µg/ml Brefeldin A (Sigma, St Louis, MO) for 5 h. Intracellular cytokine staining was performed using BD Cytofix/Cytoperm Buffer (BD Biosciences) according to the manufacturer’s recommended procedures after surface staining of CD3, CD4 and CD8. The cells were then analyzed with FACSAria and the data with FlowJo software.

## Results

### Patient characteristics and safety assessment

This study’s enrolment comprised 14 patients with a median age of 48 years, all of whom were WT1 vaccinated (Fig. 1). Twelve of them had glioblastoma multiforme (GBM) and two had anaplastic astrocytoma. These malignant gliomas were refractory to temozolomide. To prevent brain edema, ten patients with neurological symptoms were treated with steroids, while the remaining four patients with PS 0 were not given steroids.

Three of the patients were vaccinated with 0.75 mg, four with 1.5 mg, and four with 3.0 mg of WT1 HLA class II peptide, as well as with 3.0 mg of WT1 HLA class I peptide for all 14 subjects. All vaccinations were completed in 6 weeks (Table 1). Evaluation for safety showed that 11 patients did not have DLT as the primary endpoint, while three patients dropped out within 6 weeks due to disease progression. Grade I skin eruption was observed at the injection sites in all patients, but no grade III/IV hematological or non-hematological toxicities were detected for any dosage of WT1 HLA class II peptide during the 6-week period.

**Table 1:** Patients’ characteristics

Patient no.	Age	Sex	Diseases	Operation	MGMT methylation	Pretreatment	Status before WT1 vaccine			
							CR/relapse	Dissemination	Steroid	PS
1	45	F	GBM	Resection	N.D	RT/TMZ, CARE	Relapse	(−)	(−)	1
2	39	F	GBM	Resection	N.D	RT/TMZ, ACNU	Relapse	(−)	(+)	1
3	61	M	GBM	Resection	N.D	RT/TMZ	Relapse	(−)	(+)	1
4	64	F	GBM	Resection	N.D	RT/TMZ, IFN	Relapse	(−)	(−)	1
5	55	F	GBM	Resection	N.D	RT/TMZ, PAV	Relapse	(−)	(−)	0
6	58	M	GBM	Resection	(+)	RT/TMZ	Relapse	(−)	(−)	0
7	31	M	A.A	–	N.D	RT/TMZ	Relapse	(−)	(−)	0
8	55	F	GBM	Resection	N.D	RT/TMZ	Relapse	(−)	(+)	1
9	38	M	A.A	–	(−)	RT/TMZ, CARE	Relapse	(−)	(−)	1
10	44	M	GBM	Resection	N.D	RT/TMZ	Relapse	(−)	(+)	1
11	53	M	GBM	Resection	(−)	RT/TMZ	Relapse	(−)	(−)	0

*GBM* glioblastoma multiforme, *AA* anaplastic astrocytoma, *MGMT* O6-methylguanine-DNA methyltransferase, *N.D* not determined, *RT* radiation therapy, *TMZ* temozolomide, *IFN* interferon, *ACNU* nimustine hydrochloride, *CARE* carboplatin and etoposide, *PAV* procarbazine, ACNU and vincristine, *PS* ECOG performance score

### Clinical outcomes

**Fig. 2:**
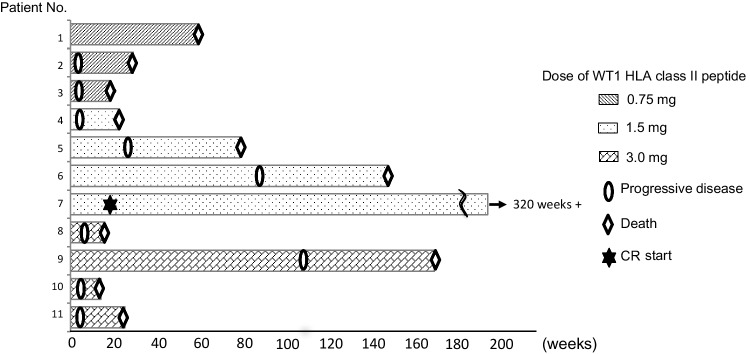
Schema for WT1 tetramer and intracellular cytokine assays. PBMC samples were divided in two, and one part was used for the WT1 tetramer assay. The other part was cultured in the presence of WT1 HLA class I and II peptides, and IL-2 for 1 week and then used for the WT1 tetramer and intracellular cytokine assays after re-stimulation with each of the WT1 peptides

Six of the 14 patients had stable disease 6 weeks after WT1 vaccination. The median OS time and 1-year OS rate were 24.7 weeks and 36%, respectively (Table 2). Figure 2a shows swimmer plot of clinical outcomes for each patient.

**Table 2:** WT1-specific immune and clinical responses

Patient no.	Doses (mg)	Times	AE	DTH (mm) 235/332	WT1 tetramer+CD8* T cells/ CD8*T cells (%)^a^			IFN-y-producing CD8*T cells/ CD8*T cells (%)^a^			TNF-a-producing CD4^+^T cells/CD4*T cells (%)^a^			Clinical responses		Outcome	
					Pre	4–8 weeks	10 months–1 year 3 months	Pre	4–8 weeks	10 months–1 year 3 months	Pre	4–8 weeks	10 months–1 year 3 months	6 weeks	12 weeks	PFS (weeks)	OS (weeks)
1	0.75	30	(−)	*–*/3	0.03	1.25		1.3	1.05		0.33	6.72		SD	SD	58.4	58.4
2	0.75	21	(−)	–/–	0.76	43.9		0	39.8		0	48.3		PD	PD	4	28.5
3	0.75	11	(−)	4/6	0.09	2.55		0	2.2		0	26.7		PD	PD	4	18.4
4	1.5	10	(−)	–/–	0.29	0.31		0	0.03		0.33	0.39		PD	PD	4	22.2
5	1.5	35	(−)	3/–	0.05	1.06	0.14	0	2	0.68	0.87	3.7	1.96	SD	SD	23.7	77.1
6	1.5	49	(−)	–/3	0.02	2.50	0.36	0.25	12.11	2.18	0.16	12.69	8.73	SD	SD	84.5	144.5
7	1.5	63	(−)	–/–	0.04	11.6	28.00	0.16	4.79	21.33	0.04	41.4	41.5	SD	SD	> 320.1	> 320.1
8	3	g	(−)	–/–	0.03			0.27			0			SD	PD	8.8	15.7
9	3	49	(−)	–/–	0.09	0.50	0.14	0	0.15	0.2	0.17	2.34	10.23	SD	SD	105.3	165.8
10	3	7	(−)	4/–	0.1	5.42		0.23	6.08		0.01	17.21		PD	PD	4	13.9
11	3	25	(−)	3/3	0.08	2.03		0	0.53		0.04	7.52		PD	PD	4.2	24.7

*DTH* delayed-type hypersensitivity, *Doses* doses of WT1 class II peptide, *Times* times of WT1 vaccine, *AE* adverse effect, *SD* stable disease, *PD* progressive disease

^a^Frequencies of WT1 tetramer^+^ CD8^+^ T cells, IFN-γ-producing CD8^+^ T cells, and IFN-α-producing CD4^+^ T cells were analyzed after 1-week culture of PBMCs

### Immunological responses and their possible associations with clinical outcomes

**Fig. 3:**
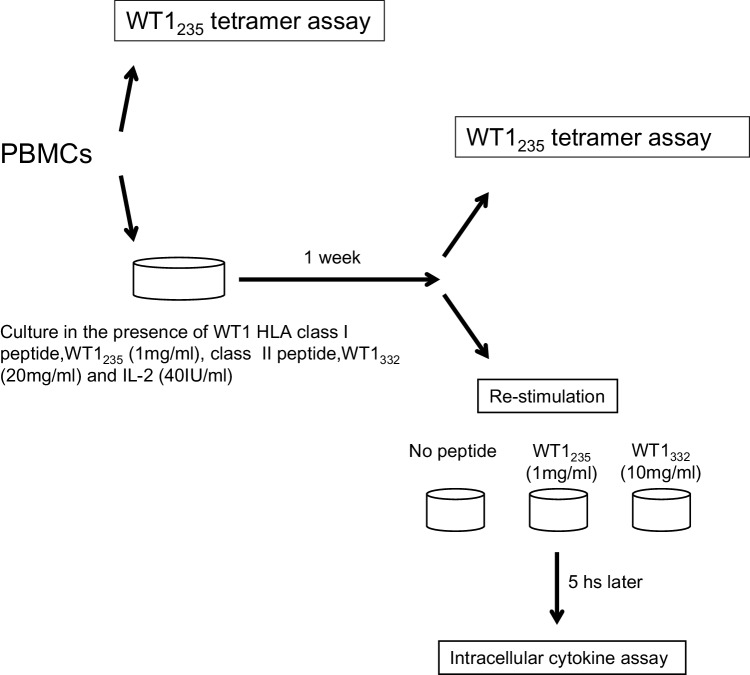
A swimmer plot of clinical outcomes for each patient

**Fig. 4:**
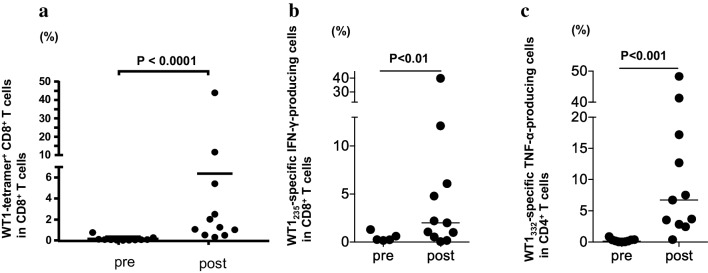
Induction of WT1-specific T cells by WT1 peptide vaccination. **a** Induction of WT1 tetramer^+^ CD8^+^ T cells by WT1 peptide vaccination. The WT1 tetramer assay was performed after culture for 1 week in the presence of WT1 HLA class I and II peptides, and IL-2. Frequencies of WT1 tetramer^+^ CD8^+^ T cells in CD8^+^ T cells after culture for 1 week are shown for each patient pre- and 4–8 weeks after WT1 vaccination. **b, c** Analysis of WT1 HLA class I-specific cytokine-producing CD8^+^ T cells and class II peptide-specific cytokine-producing CD4^+^ T cells. Frequencies of WT1 HLA class I (WT1_235_)-specific IFN-γ-producing CD8^+^ T cells (left) and WT1 HLA class II (WT1_332_)-specific TNF-α-producing CD4^+^ T cells (right) were examined by means of re-stimulation with each WT1 peptide after a 1-week culture in the presence of WT1 HLA class I (WT1_235_) and II (WT1_332_) peptides, and IL-2. The frequencies are shown for each patient pre- and 4–8 weeks post-WT1 vaccination

Frequency of WT1-specific CD8^+^ T cells in PBMCs was assayed using the WT1 tetramer, once just after sampling and again after the culture for 1 week, in the presence of WT1 HLA class I and II peptides, and of IL-2 (Fig. 3). Frequency of WT1-specific CD8^+^ T cells after the culture for 1 week is shown in Fig. 4a and Table 2 for each patient both before WT1 vaccination and 4–8 weeks after WT1 vaccination. WT1 tetramer^+^ CD8^+^ T cells had markedly increased 4–8 weeks after WT1 vaccination in 7 of the 11 patients who had completed the 6-week WT1 vaccination schedule.

WT1 HLA class I peptide (WT1_235_)-specific IFN-γ-producing CD8^+^ T cells had increased markedly in 6 of the 11 patients (Fig. 4b), while WT1 HLA class II peptide (WT1_332_)-specific TNF-α-producing CD4^+^ T cells showed a marked increase in 10 of 11 patients 4–8 weeks after WT1 vaccination (Fig. 4c).

These results demonstrated that both WT1 HLA class I peptide (WT1 _235_)-specific CD8^+^ T cells and WT1 HLA class II peptide (WT1_332_)-specific CD4^+^ T cells were induced by the cocktail vaccine of WT1 HLA class I and II peptides.

DTH of all patients for WT1 HLA class I and II peptides was negative before WT1 peptide vaccination, but in 6 weeks had become positive for WT1 HLA class I peptide alone in two, for WT1 class II peptide alone in two, and for both WT1 HLA class I and II peptides in two among 11 patients (Table 2).

**Fig. 5:**
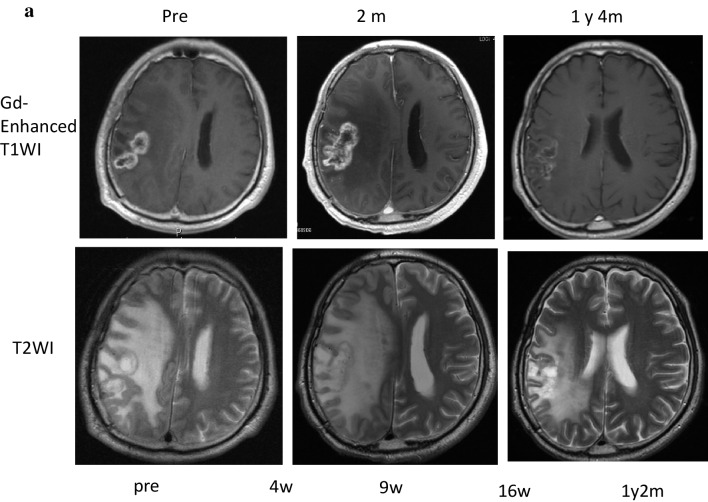

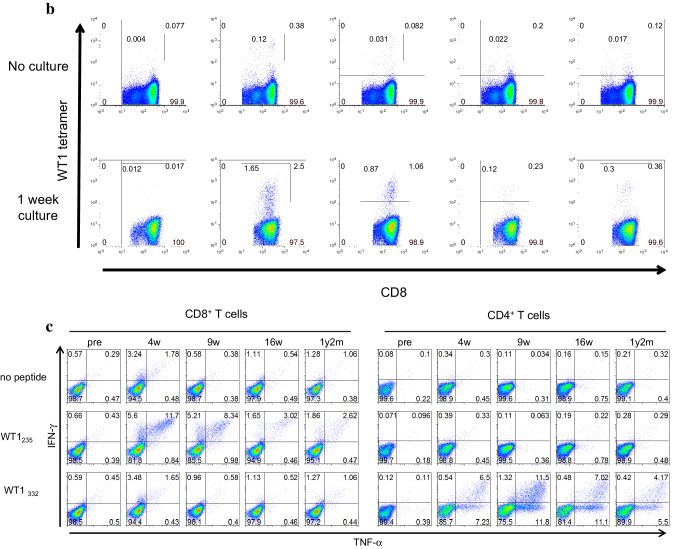
Clinical course and immune-monitoring of patient 6. **a** Magnetic resonance imaging (MRI) results are shown at three time points: pre-, 2 months (2 m) and 1 year and 4 months (1 y 4 m) post-WT1 vaccination. Upper and lower columns show Gadolinium (Gd)-enhanced weighted imaging (Gd-enhanced WI) and T2 weighted imaging (T2WI), respectively. FACS analysis for WT1 tetramer FACS analysis for WT1 tetramer^+^ CD8^+^ T cells. Dot blots of WT1 tetramer^+^ CD8^+^ T cells are shown pre- and at 4 weeks, 9 weeks, 16 weeks, and 1 year and 2 months post-WT1 vaccination. Upper and lower columns show tetramer assay without and with 1-week culture of PBMCs, respectively. **c** FACS analysis of cytokine-producing CD8^+^ and CD4^+^ T cells. Dot blots of IFN-γ- and/or -TNF-α-producing CD8^+^ and CD4^+^ T cells are shown pre- and at 4 weeks, 9 weeks, 16 weeks, and 1 year and 2 months post-WT1 vaccination. PBMCs were not re-stimulated or re-stimulated with either WT1_235_ or WT1_332_ peptide and analyzed

The clinical course and immune-monitoring of patient 6 with GBM in the right parietal lobe are shown in Fig. 5. The patient received partial resection of the GBM, followed by combination therapy of radiation and temozolomide (TMZ), and two subsequent courses of maintenance therapy with TMZ. However, since this patient was suffering from a progressive disease, he was enrolled in this clinical study and received a total of 49 WT1 vaccinations over 1 year and 9 months. His disease was kept stable for about 1 year, and after that reduction in the Gd-enhanced lesion and improvement in the midline shift on MRI were observed (Fig. 5a). After that, however, he had recurrence near the original tumor 1 year and 9 months after the WT1 vaccination. Frequency of WT1 tetramer^+^ CD8^+^ T cells had notably increased from 0.017 to 2.5% 4 weeks after the WT1 vaccination and a high frequency of 0.36% remained for 1 year and 2 months after the vaccination (Fig. 5b).

CD8^+^ and CD4^+^ T cells of patient 6 produced both interferon gamma (IFN-γ) and tumor necrosis factor alpha (TNF-α) in response to WT1 HLA class I (WT1_235_) and II (WT1_332_) peptides, respectively, both time at 4 weeks, and 1 year and 2 months post-WT1 vaccination (Fig. 5c).

All four patients who survived over 1 year maintained high frequencies of WT1-specific CD8^+^ T cells and Th1-typed CD4^+^ T cells during about 1 year of WT1 vaccination (Table 2).

## Discussion

This is the first report of use of a cocktail vaccine of WT1 HLA class I and II peptides for the treatment of malignancies. In this phase I study, we evaluated first the safety and second the efficacy of WT1 peptide vaccine for patients with recurrent malignant glioma. Adverse events of grade III/IV were not observed and this therapy was well tolerated at any dose of the WT1 HLA class II peptide. Thus, the safety of the cocktail vaccine of WT1 HLA class I and II peptides was confirmed.

We previously treated hematopoietic malignancies including leukemia and various kinds of solid tumors with WT1 HLA class I peptide vaccine and reported that the vaccination elicited WT1-specific immunological response, followed by clinical responses without severe adverse effects [29]. One of the most important problems of WT1 vaccination with WT1 HLA class I peptide alone was that WT1-specific CD8^+^ T cells generally increased at 4–8 weeks post-WT1 vaccination, but afterwards decreased gradually, and their frequency could not be maintained for a long time (data not shown). It should be noted that, compared with WT1 vaccination with the WT1 HLA class I peptide alone, a cocktail vaccine with WT1 HLA class I and II peptides tended to result in a powerful induction of WT1-specific CD8^+^ T cells 4–8 weeks after WT1 vaccination and in long-term maintenance of a high frequency of WT1-specific CD8^+^ T cells (> 0.1%) and Th1-typed WT1-specific CD4^+^ T cells for about 1 year, as observed in four long-term survivors who were treated with the cocktail vaccine (Table 2).

We previously reported that WT1_332_ peptide in vitro could induce WT1_332_-specific Th1-type CD4^+^ T cells which caused an increase in WT1-specific CD8^+^ CTLs [24]. In the study reported here, the cocktail vaccine of WT1 HLA class I and II peptides in vivo induced, as expected, WT1 HLA class I peptide (WT1_235_)-specific CD8^+^ CTLs and class II peptide (WT1_332_)-specific IFN-γ-producing Th1-type CD4^+^ T cells. We assume that the induction of WT1 class II peptide-specific Th1-type CD4^+^ T cells generated the strong and long-term maintenance of WT1-specific CD8^+^ T cells.

Dendritic cell (DC) vaccines using WT1 HLA class I and II peptides (DC/WT1-I/II) have recently been administered by several institutes, and it was reported that they were well tolerated and induced WT1-specific immune responses and clinical effects [30–33]. Homma et al. performed a phase I clinical study (DC/WT1-I/II) of treatment for patients with pancreatic ductal adenocarcinoma using DCs pulsed with a mixture of the same WT1 HLA class I and II peptides as those used in our study, combined with chemotherapy [30]. They demonstrated that the combination therapy was well tolerated and WT1-specific IFN-γ-producing CD4^+^ T cells increased significantly following treatment with DC/WT1-I/II. WT1 peptide-specific DTH was detected in four of the seven patients vaccinated with DC/WT1-I/II while the WT1-specific DTH-positive patients showed a significant improvement in OS and progression-free survival (PFS) compared with the historical control (chemotherapy only) patients [30]. Safety, enhanced immunological responses, and favorable clinical outcomes observed in our study seem to be consistent with the results of previous studies of these DC vaccine therapies.

The data accumulated so far demonstrate that WT1 vaccine induce clinical effects in association with the induction of WT1-specific CTLs, but do not cause damage to WT1-expressing normal tissues. The question of why the WT1 vaccine does not damage WT1-expressing normal cells is very important. Two possible explanations are that, first, normal WT1-expressing cells are ignored because the expression of WT1 peptide/MHC complexes on the cell surface is too little to be recognized by WT1-specific CTLs. The second explanation is that normal WT1-expressing cells possess as yet unknown protective mechanisms against WT1-specific CTL-mediated cytotoxicity. Although the cocktail vaccine used in our study induced stronger WT1-specific CTL responses than those induced by WT1 HLA class I peptide alone, no toxicity for normal tissue was observed, which is an important point for the use of this vaccine in clinical settings.

## Abbreviations

AML: Acute myeloid leukemia
CTL: Cytotoxic T lymphocyte
DLT: Dose-limiting toxicity
DTH: Delayed-type hypersensitivity
FACS: Fluorescence-activated cell sorting
GBM: Glioblastoma multiforme
HLA: Human leukocyte antigen
IFN: Interferon
mAbs: Monoclonal antibodies
MRI: Magnetic resonance imaging
OS: Overall survival
PBMCs: Peripheral blood mononuclear cells
TAA: Tumor-associated antigen
Th1: T-helper cell type 1
TMZ: Temozolomide
TNF: Tumor necrosis factor
WT1: Wilms’ tumor gene 1
